# Excessive iodine induces thyroid follicular epithelial cells apoptosis by activating HIF-1α-mediated hypoxia pathway in Hashimoto thyroiditis

**DOI:** 10.1007/s11033-023-08273-z

**Published:** 2023-02-17

**Authors:** Lili Zhang, Xiaojing Sun, Lin Liu, Ping Wang, Linxue Qian

**Affiliations:** 1 Department of Ultrasound, Beijing Friendship Hospital, Capital Medical University, Beijing, China; 2 Department of International Medical Center, Beijing Friendship Hospital, Capital Medical University, Beijing, China; 3 Department of Liver Research Center, Beijing Friendship Hospital, Capital Medical University, Beijing, China

**Keywords:** Hashimoto’s thyroiditis, Apoptosis, HIF-1α, Thyroid follicular cell

## Abstract

**Background:**

Hashimoto thyroiditis (HT) is considered the most common autoimmune thyroid disease. A growing body of evidence suggests that HT incidence correlates with excessive iodine intake. We should probe the effects of excessive iodine intake in HT development and its possible mechanism.

**Methods and results:**

The study recruited 20 patients: 10 with HT and 10 with nodular goiter. We detected the expression of an apoptosis-related protein caspase-3 by immunohistochemistry. In vitro study, we explored the proliferation and apoptosis status in thyroid follicular cells (TFCs) stimulated with different iodine concentrations by MTT and flow cytometry. Then we performed RNA sequence analysis of Nthy-ori3-1 cells treated for 48 h with KI to probe the underlying mechanism. Finally, we used RT-PCR and siRNA interference to verify the results. We identified apoptosis in thyroid tissue obtained from HT patients coincides with the increase of caspase-3 levels. In vitro study, iodine suppressed proliferation of TFCs and promoted TFCs apoptosis in a dose-dependent manner with regulating caspase-3 activation. HIF-1α-NDRG1 mediated hypoxia pathway activation promoted the transmission of essential apoptosis signals in TFCs.

**Conclusion:**

Our study confirmed that excessive iodine adsorption activates the HIF-1α-mediated hypoxia pathway to promote apoptosis of TFCs, which may be an important risk factor contributing to HT development.

## Background

It is well-known that iodine is an essential micronutrient for thyroid hormone synthesis, which undoubtedly plays a pivotal role in thyroid disease development. Mounting evidence suggests that severe iodine deficiency causes goiter and hypothyroidism, while excess iodine intake causes autoimmune thyroid disorders, including Hashimoto thyroiditis (HT) and Graves disease [1, 2]. The increasing incidence of HT after the implementation of universal salt iodization supports these observations [2–4]. Furthermore, patients are prone to develop hypothyroidism and papillary thyroid carcinoma when exposed to excessive iodine [5].

HT is one of the most common autoimmune thyroid diseases, accounting for 5% of the worldwide population [6]. The main features of HT are chronic lymphocytic infiltration, varying degrees of thyroid follicular cells (TFCs) atrophy, and elevated titers of thyroid autoantibodies, mainly to thyroid peroxidase antibody (TPOAb) and thyroglobulin antibody (TgAb). It is now well established that genetic susceptibility and environmental factors inducing autoimmune disorder, including the overactivation of CD4 + T cells and reduction of T regulatory cells, results in the development of HT [7, 8]. However, some studies have found that excessive iodine induces TFCs apoptosis, necrosis, and pre-infiltration of lymphocytes [9]. Thus, indicating that TFCs apoptosis preceding immune activation may be vital to cause HT.

Various internal and external stimuli could activate TFCs apoptosis, including DNA damage, the release of proinflammatory cytokines, and the aberrant expression of proapoptotic proteins [8]. Furthermore, a high number of apoptosis-related proteins levels changes during the development of HT like tumor necrosis factor-related apoptosis-inducing ligand (TRAIL), Fas ligand, caspases, and B-cell lymphoma (Bcl-2) [10, 11]. A previous study demonstrates that excessive iodine promotes TFCs apoptosis by suppressing autophagy, together with high levels of caspase-3 in HT disease. However, until now, the mechanism of excessive iodine-induced TFCs apoptosis during HT development is unclear.

According to recent research, hypoxia inducible factor-1 (HIF-1) is an important transcription factor that mediates cells adaptive response under hypoxia conditions. HIF-1 consists of HIF-1α and HIF-1β subunits, and HIF-1α is the active subunit involved in the regulation of metabolism, angiogenesis, cell proliferation, and apoptosis [12]. HIF-1α could also regulate the transcription of many apoptosis-related genes such as survivin, Bcl-2, and caspase-3 [13, 14]. This is in agreement with a recent study concluding that HIF-1α can trigger hypoxia-induced apoptosis of alveolar epithelial cells during pulmonary fibrosis development [13]. Thus far, no studies have clarified the relationship between iodine, HIF-1α, and apoptosis of thyroid follicular cells. Our study found that excessive iodine activated HIF-1α-mediated hypoxia pathway and upregulated the expression of N-myc downregulated gene-1(NDRG1), promoting TFCs apoptosis. These findings indicate that excessive iodine is a precipitant in HT development, and HIF-1α may be a potential biomarker of thyrocyte injury in HT.

## Methods

### Cell line and samples

Thyroid follicular epithelial cell line Nthy-ori3-1 (Fuheng Biotechnology Co, Ltd.) was cultured in RPMI 1640 medium (Gibco, USA) supplemented with 10% fetal bovine serum (Gibco, USA) and 2 mM L-glutamine (Biological Industries, Kibbutz Beit Haemek, Israel). The thyroid tissues used in this study were obtained from 10 patients with HT and 10 with nodular goiter. HT was diagnosed according to clinical evaluation and pathological criteria, as previously described [15]. Nodular goiter was diagnosed according to the corresponding clinical and laboratory tests. All samples were collected from the Beijing Friendship Hospital affiliated to Capital Medical University. This study was approved by the Ethics Committee of the Beijing Friendship Hospital affiliated to Capital Medical University. All participants signed the informed consent.

### Cell treatments

We achieved different concentrations of iodine stimuli by diluting potassium iodide (KI) with cell culture media to a final concentration of 20–80 mM. Nthy-ori-3 cells were treated with 20–80 mM KI for 48 h. The control group only consisted of untreated Nthy-ori-3 cells.

### Immunohistochemistry staining

Samples were fixed in 10% neutralized formalin, embedded in paraffin, and sectioned with a 4–5 μm thickness. After deparaffinization and rehydration, antigen retrieval was performed by boiling the samples in EGTA Antigen Retrieval solution (pH 8.0) and then washing the sections with phosphate-buffered saline (PBS). Thyroid sections were incubated with rabbit anti-human HIF-1α (Zhongshan Golden Bridge Biotechnology, China) and rabbit anti-human caspase-3 (Cell Signaling Technology, Danvers, MA, USA) at 4℃ overnight. After three washes with PBS, secondary antibodies (Maixin Biotechnology Co, Ltd.) were added to the sections and followed by counterstained with hematoxylin and observations under a microscope. All slides were reviewed by pathologists.

### Determination of cell viability

After incubating cells in six different concentrations of the working solutions for 48 h, the media was replaced with 200 µl of fresh media and 20 µl of MTT (5 mg/ml). After another 4 h, 150 µl DMSO was added, followed by shaking the plate for 10 min. The optical density (OD) of each well was measured at 490 nm, utilizing a microplate reader. The results were calculated based on the formula: S = (OD treated well – OD blank)/(OD control well − OD blank) × 100%.

### Analysis of apoptosis by flow cytometry

TFCs apoptosis was measured using flow cytometry (FCM), according to the annexin V-PE/7-AAD kit (BD Bioscience, San Jose, CA, USA) protocol. When the cells had grown to 80% after 48 h, Nthy-ori3-1 cells were collected and then washed with PBS, resuspended in binding buffer, and incubated with annexin V-PE/7-AAD. We used FlowJo.7.6 software to analyze the FCM results. Approximately 1 × 10^4^ cells were analyzed each sample. Results were expressed as mean ± standard deviation (SD) of three independent experiments.

### RNA isolation and reverse transcription-polymerase chain reaction (RT-PCR) analysis

After the cells were treated with KI for 48 h, we extracted the total RNA using TRIzol reagent and examined its purity. We then performed RT-PCR to detect mRNA expression levels, as previously described [16]. The following primers were synthesized by Beijing Tianyi Huiyuan Biotechnology Co. Ltd. (Beijing, China) and detailed in Table 1.

**Table 1:** Primers used in this study

Gene			Sequence (5’→3’)	
HIF-1α	Human	Sense		ATCCATGTGACCATGAGGAAATG
		Anti-sense		TCGGCTAGTTAGGGTACACTTC
Caspase-3		Sense		CATGGAAGCGAATCAATGGACT
		Anti-sense		CTGTACCAGACCGAGATGTCA
NDRG1		Sense		GTCCTTATCAACGTGAACCCTT
		Anti-sense		GCATTGGTCGCTCAATCTCCA
GAPDH		Sense		GGAGCGAGATCCCTCCAAAAT
		Anti-sense		GGCTGTTGTCATACTTCTCATGG

### RNA sequence analysis

Nthy-ori3-1 cells were treated with or without KI (40 mM) for 48 h. Then total RNA was extracted using TRIzol reagent for RNA sequence analysis (Zhejiang Annuoyouda Biotechnology, China). Then the data was analyzed using GSEA.

### siRNA treatment

Nthy-ori3-1 cells were transfected with *HIF-1a*-specific siRNAs or non-targeting control (scrambled) siRNAs (GenePharma, Shanghai, China), according to standard protocols. Briefly, the cells were cultured in 24-well plates with 500 µl RPMI 1640 basal culture medium containing 10% fetal bovine serum, until a confluence of 70- 80% was obtained. To prepare the transfection complex, Lipofectamine RNAiMAX transfection reagent (Thermo Fisher Scientific, Waltham, MA, USA; 1 µL per well) was incubated with the *HIF-1a* siRNAs or the scrambled siRNA (2 µL per well) in antibiotic-free and serum-free medium for 20 min at room temperature. The cells were then incubated with siRNA-Lipofectamine RNAiMAX complex at 37 °C for 24 h. After 24 h, the transfection mixture was replaced and the cells were stimulated with or without KI for 48 h. The apoptosis, active caspase-3 (GreenNuc™ Caspase-3 Assay Kit for live cells, C1168M, Shanghai, China) and NDRG1 expression (26902-1-AP, Proteintech, USA) were further examined using flow cytometry. The sequences of HIF-1α siRNAs and scrambled siRNAs are listed in Table 2.

**Table 2:** The sequences of HIF-1α siRNA

Gene	Strand	Sequence (5’→3’)	
Negative control			UUCUCCGAACGUGUCACGUTT
			ACGUGACACGUUCGGAGAATT
HIF-1α			GCUGGAGACACAAUCAUAUTT
			AUAUGAUUGUGUCUCCAGCTT

### Statistical analysis

All the data in this study were presented as mean ± SD and processed with SigmaPlot 12.5 software. Differences among various groups were analyzed by using one-way ANOVA. All quantitative data were analyzed using SPSS version 16.0 (SPSS, Inc., Chicago, USA). P < 0.05 was considered statistically significant.

## Results

### Increased apoptosis occurs in thyroid tissues from HT patients

**Fig. 1:**
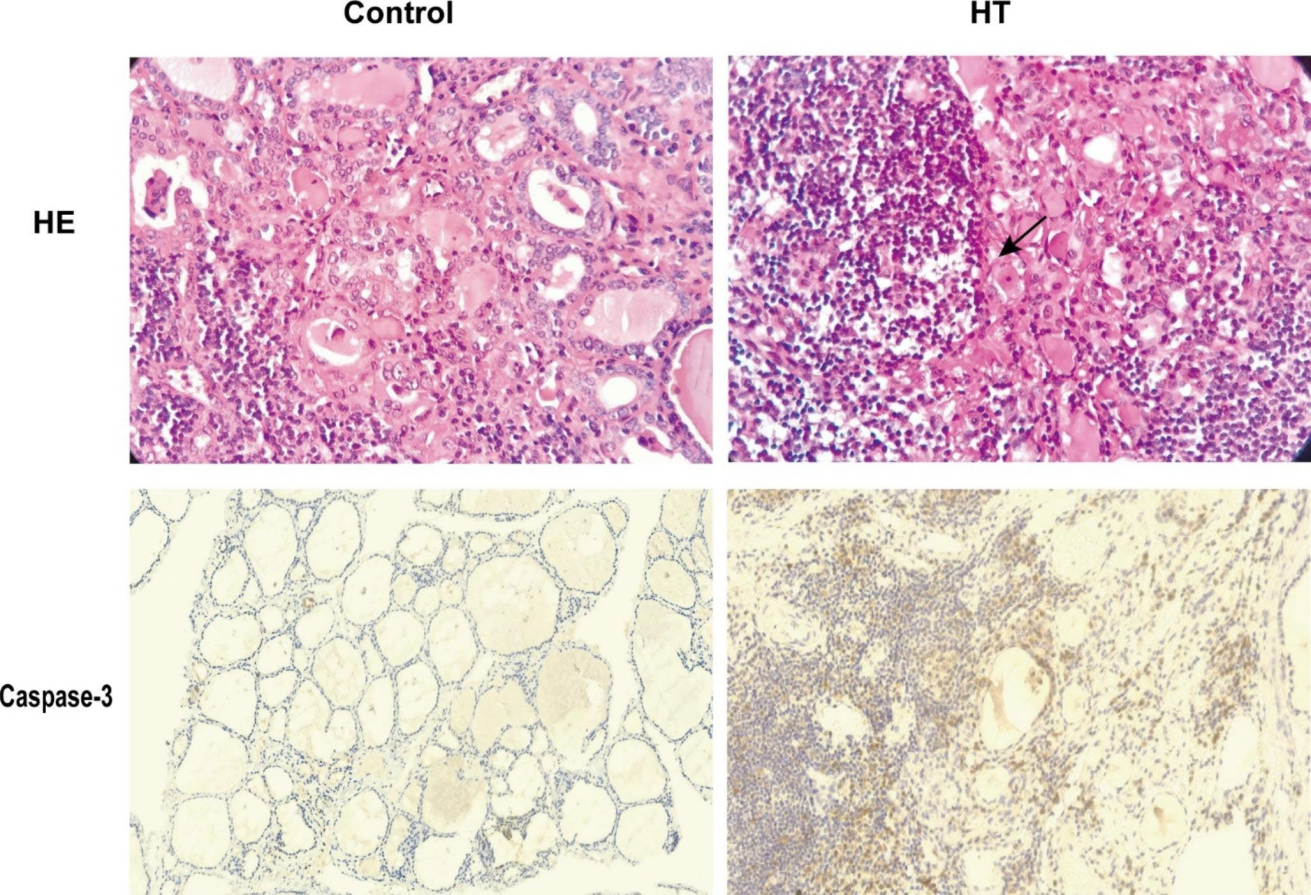
Representative image of hematoxylin-eosin (HE) staining and caspase-3 immunohistochemical staining in thyroid tissue from HT (n = 10) and control (n = 10) groups. The black arrow in the HE panel marks acidophilic degeneration in HT tissue. Brown regions represent caspase-3 positive expression (original magnification, 100×)

To assess TFC status from HT patients, we detected the expression of caspase-3 by immunohistochemistry, which is an apoptosis-related protein. The results showed that caspase-3 expression increased in thyroid tissues from HT patients (n = 10) when compared to the control group (n = 10; P < 0.05; Fig. 1; Table 3). Thus, indicating that apoptosis is elevated in thyroid tissues from HT patients.

**Table 3:** Caspase-3 expression in Hashimoto thyroiditis (HT) and control tissues

	HT n (%)	Control n (%)	P-value
Caspase-3			0.03
positive	5(50%)	0(0%)	
negative	5(50%)	5(100%)	

p < 0.05, HT group vs. control group in terms of the expression of caspase-3

### Iodine suppresses proliferation of TFCs in a dose-dependent manner

**Fig. 2:**
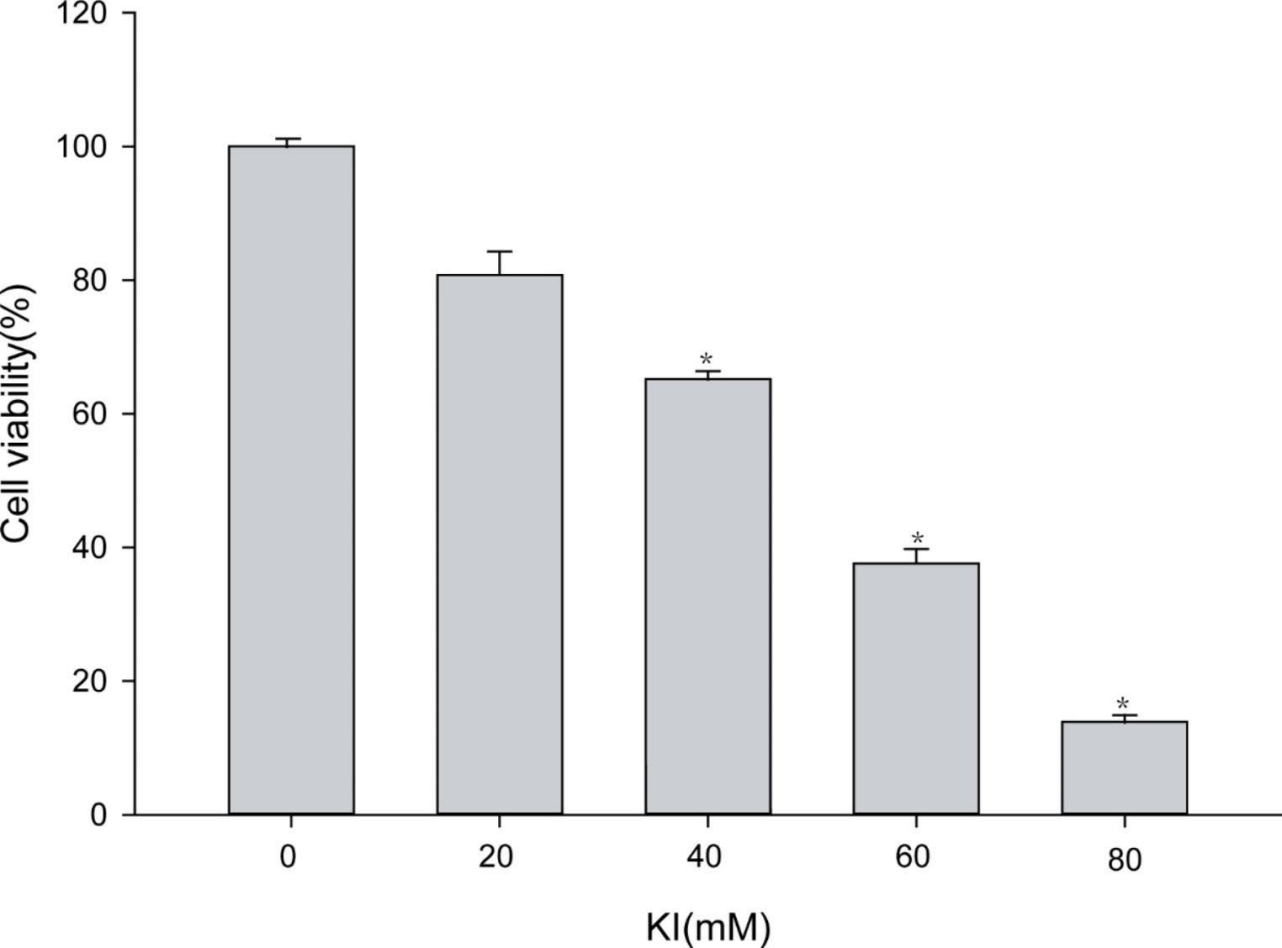
The effect of iodine in Nthy-ori3-1 thyroid cell line viability. MTT assay results of TFCs treated for 48 h with different KI concentrations, indicating that iodine caused a concentration-dependent decrease in the cell viability of Nthy-ori3-1 thyroid cell line. The bar graphs represent the means ± SD from 3 independent experiments. *P < 0.05 compared to the control group

Iodine caused concentration-dependent cell injury in the Nthy-ori3-1 thyroid cell line (Fig. 2). After Nthy-ori-3 cells were treated with different concentrations of KI for 24 h, we measured cell viability of all groups by MTT assay. We found that the cell viability in the 40mM, 60mM, and 80mM KI-treated groups were remarkably lower than that of the control group (P < 0.05). Moreover, the cell viability in the group treated with 80mM KI was significantly lower than the group treated with 40mM KI (P < 0.01).

### Excessive iodine promotes TFCs apoptosis

**Fig. 3:**
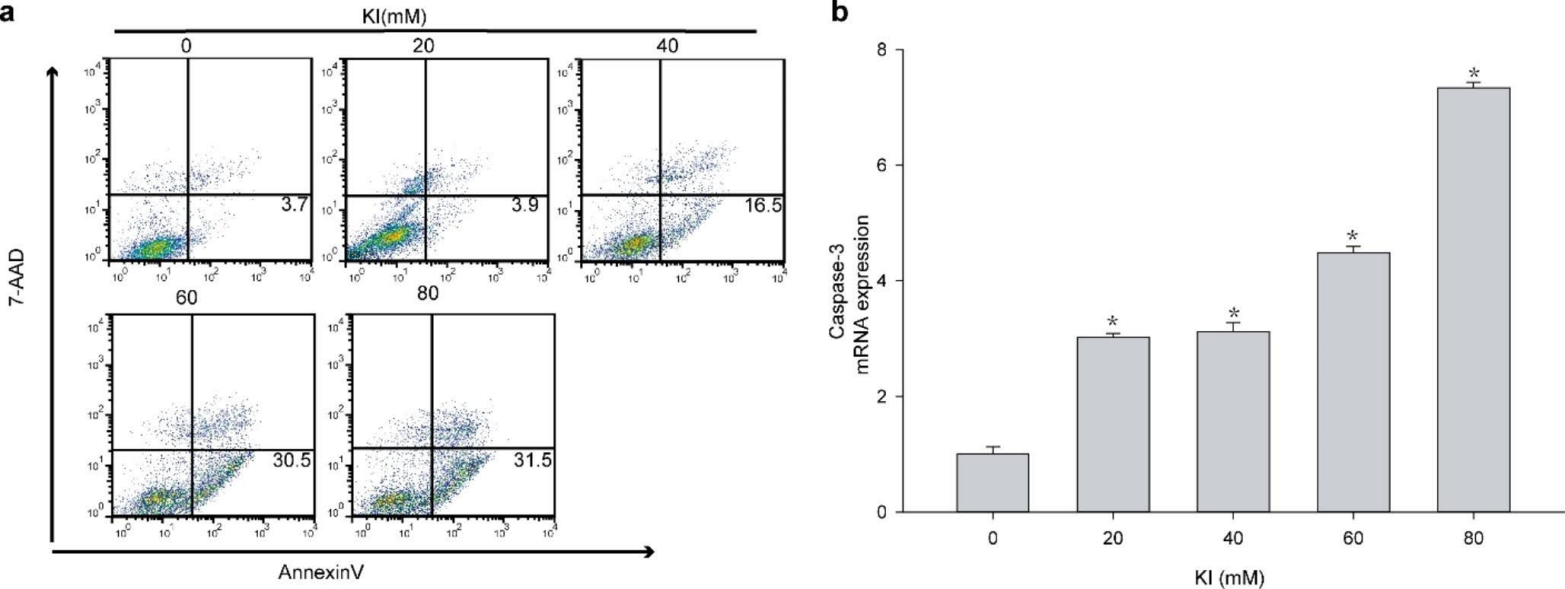
The effect of iodine in Nthy-ori3-1 thyroid cell line apoptosis. **a** After thyroid follicular epithelial cells were treated with different concentrations of KI for 48 h, TFCs apoptosis was measured by flow cytometry. Apoptosis rates of KI-treated TFCs increased significantly in a dose-dependent manner. **b** Caspase-3 mRNA expression increased in the KI-treated groups compared to the control group. The bar graphs represent the means ± SD from 3 independent experiments. *P < 0.05 compared to the control group

Apoptosis of TFCs was measured in Nthy-ori3-1 cells incubated with different KI concentrations for 48 h by annexin V-PE/7-AAD staining. We found that apoptosis rates in KI-treated TFCs increased markedly in a dose-dependent manner (Fig. 3a). Simultaneously, caspase-3 mRNA expression increased in KI-treated groups when compared to the control group. The 80mM KI-treated group also showed substantial TFCs apoptosis than other groups (P < 0.05, Fig. 3b), suggesting that excessive iodine absorption promotes TFCs apoptosis.

### Excessive iodine promoted TFC apoptosis survival via the HIF-1α-NDRG1 mediated hypoxia pathway

**Fig. 4:**
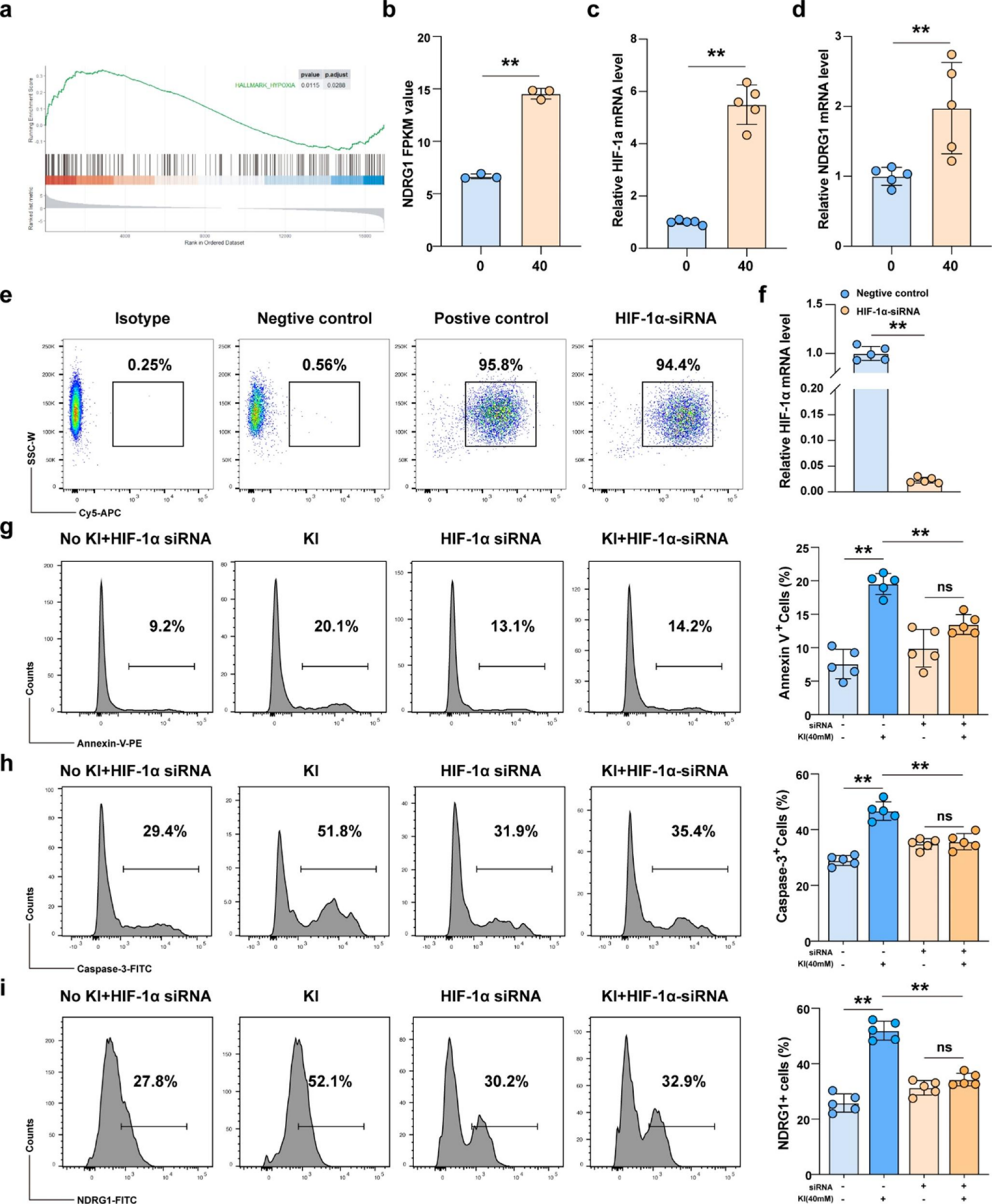
HIF-1α mediated hypoxia pathway was activated by excessive iodine stimulation. **a** RNA sequence analysis suggested that the hypoxia pathway was significantly activated in TFCs with KI stimulation. **b** FPKM value from RNA sequencing showed that NDRG1 associated with hypoxia pathway was upregulation in TFCs with KI stimulation. **c** RT-PCR results showed that *HIF-1α* mRNA expression was increased with KI stimulation. **d** RT-PCR results showed that *NDRG1* mRNA expression was increased with KI stimulation. The efficiency of Cy5-labeled *HIF-1α* siRNA interference was examined through flow cytometry (**e**) and RT-PCR (**f**) in TFCs. Representative flow cytometry plots (left) and statistical analysis (right) of Annexin V+ (**g**), caspase-3+ (**h**), and NDRG1+ (**i**) cells relative to TFCs with or without siRNA interference and KI treatment. Data are presented as mean ± SD; **P < 0.01; ns, no significance

To further explore the molecular mechanism underlying KI-induced cell apoptosis, we performed RNA sequencing of Nthy-ori3-1 cells with or without KI stimulation (40mM for 48 h). GSEA showed that the hypoxia pathway was increased with KI stimulation (Fig. 4a) and accompanied with upregulation of NDRG1 expression (Fig. 4b). We further confirmed the *HIF-1α* and *NDRG1* mRNA expression by RT-PCR in Nthy-ori-3 cells treated with 40mM KI for 48 h. The results showed that KI stimulation of TFCs resulted in an increase in expression of *HIF-1α* and *NDRG1* (Fig. 4c and d). To further confirm the role of HIF-1α in KI regulation of TFCs, we used fluorescent dye (Cy5)-labeled siRNA to knockdown HIF-1α expression. As shown in Fig. 4e and f, flow cytometry and RT-PCR analysis indicated that siRNA transfection significantly inhibited HIF-1α expression in TFCs compared with the scramble siRNA-transfected cells. Our experiments showed that KI stimulation promoted TFCs apoptosis, caspase-3 activation and NDRG1 expression. However, KI regulation in these cells was impaired after HIF-1α knockdown (Fig. 4 g, Fig. 4 h and Fig. 4i). Overall, these results suggest that excessive iodine intake could activate the HIF-1α-mediated hypoxia signaling pathway and upregulate NDRG1 expression, promoting TFCs apoptosis.

## Discussion

HT is an organ-specific autoimmune disease that has a genetic predisposition. Despite being an essential microelement for thyroid hormone synthesis, iodine also can contribute directly to the development of thyroid disorders, such as thyroid autoimmunity [17]. To our knowledge, the present study is the first to demonstrate that excessive iodine intake promotes TFCs apoptosis, possibly by activating HIF-1α-mediated hypoxia signaling pathways and upregulating NDRG1 expression.

Apoptosis plays a vital role in HT development. For instance, M. Erdogan found that the atrophy and destruction of thyroid follicles were connected with Fas-mediated apoptosis in HT patients [18]. Fas combined with Fas ligand (FasL) activates caspase-8 induced cysteine proteases caspase-3 complex, which selectively affects some proteins activity and leads to TFCs apoptosis. Moreover, activated caspases-8 initiates the intrinsic apoptotic pathway by Bcl-2 [8]. Therefore, caspase-3 is involved in Fas-mediated apoptosis as a key protease. In our study, we found that caspase-3 expression in HT patients was upregulated compared with that of controls. This result agrees with recent reports demonstrating high caspase-3 levels in HT patients [9]. While investigating the effects of excessive iodine on TFCs apoptosis in vitro, we observed that iodine suppressed TFCs proliferation in a dose-dependent manner and promoted the expression of apoptosis-related proteins. Similarly, the expression of caspase-3 in HT tissue also increased, indicating that excessive iodine may promote HT development.

Nevertheless, the results of this study suggest that iodine could induce HT development by activating the apoptosis signaling pathway in TFCs, the molecular mechanism behind this process was unclear. Furthermore, there was evident activation of the hypoxia signaling pathway under high KI concentrations. PCR analysis from in vitro experiments corroborated that the expression of HIF-1α and caspase-3 in iodine-treated Nthy-ori3-1 cells increased along with increased concentrations. Therefore, we speculate that excessive iodine may induce TFCs apoptosis through the activation of the HIF-1α-mediated pathway.

Under hypoxic conditions, HIF-1α could bind to HIF-1β to form the HIF-1 complex. Consequently, this complex formation could activate gene expression by binding to hypoxia response elements (HREs) located in the promoter region of some genes such as NDRG1 [19–21]. The *NDRG1* gene is a member of the N-myc downregulated genes and encodes the NDRG1 protein. This protein consists of 394 amino acids and is widely expressed in the cytoplasm of different cell types. NRDG1 is mainly involved in apoptosis, proliferation, migration, and angiogenesis processes [22]. A recent study showed that high NDRG1 expression levels resulted from the activation of the HIF-1 signal during hypoxia [23]. On the other hand, there is strong evidence showing that the NDRG1 gene possessed two HREs located at -1376 and − 7503 bp. Thus, HIF-1 could induce NDRG1expression by binding to its HREs [24]. Our in vitro PCR analysis showed that the expression of NDRG1 in iodine-treated Nthy-ori3-1 cells is proportionally increased. Taken together, these findings suggest that excessive iodine may activate the HIF-1α-mediated hypoxia signaling pathway and upregulate expression of NDRG1, therefore promote apoptosis of TFCs and the development of HT. Furthermore, we also confirmed the role of HIF-1α in KI regulation of TFCs with siRNA interference, and we found that KI regulation in TFCs was impaired after HIF-1α knockdown and accompanied by NDRG1 downregulation. HIF-1α-NDRG1 mediated hypoxia pathway activation promoted the transmission of essential apoptosis signals in TFCs.

## Conclusion

In conclusion, our study confirmed that excessive iodine adsorption activates the HIF-1α-mediated hypoxia pathway in the TFCs apoptosis process. An increased apoptosis rate was also observed in HT patients. These results suggest that excessive iodine may promote the development of HT by inducing TFCs apoptosis. The present study raises the possibility that HIF-1α may be a potential biomarker of thyrocyte injury in HT. Therefore, we should take up appropriate iodine to reduce the prevalence of HT.

## Data Availability

All data generated or analyzed during this study are included in this published article.
