# Correction to: Excessive iodine induces thyroid follicular epithelial cells apoptosis by activating HIF-1α-mediated hypoxia pathway in Hashimoto thyroiditis

**DOI:** 10.1007/s11033-023-08351-2

**Published:** 2023-04-08

**Authors:** Lili Zhang, Xiaojing Sun, Lin Liu, Ping Wang, Linxue Qian

**Affiliations:** 1 Department of Ultrasound, Beijing Friendship Hospital, Capital Medical University, Beijing, China; 2 Department of International Medical Center, Beijing Friendship Hospital, Capital Medical University, Beijing, China; 3 Department of Liver Research Center, Beijing Friendship Hospital, Capital Medical University, Beijing, China

**Correction to: Molecular Biology Reports** 10.1007/s11033-023-08273-z

In the published article, the statement “Lili Zhang and Xiaojing Sun contributed equally to this work” was missed.

The original version of the article has been corrected.

